# Serologic Surveillance for SARS-CoV-2 Infection among Wild Rodents, Europe

**DOI:** 10.3201/eid2812.221235

**Published:** 2022-12

**Authors:** Vincent Bourret, Lara Dutra, Hussein Alburkat, Sanna Mäki, Ella Lintunen, Marine Wasniewski, Ravi Kant, Maciej Grzybek, Vinaya Venkat, Hayder Asad, Julien Pradel, Marie Bouilloud, Herwig Leirs, Valeria Carolina Colombo, Vincent Sluydts, Peter Stuart, Andrew McManus, Jana A. Eccard, Jasmin Firozpoor, Christian Imholt, Joanna Nowicka, Aleksander Goll, Nathan Ranc, Guillaume Castel, Nathalie Charbonnel, Tarja Sironen

**Affiliations:** University of Helsinki Medicum and Veterinary Medicine, Helsinki, Finland (V. Bourret, L. Dutra, H. Alburkat, S. Mäki, E. Lintunen, R. Kant, V. Venkat, H. Asad, T. Sironen);; Institut national de recherche pour l’agriculture, l’alimentation et l'environnement (INRAE), UR 0035 Comportement et écologie de la faune sauvage, Castanet-Tolosan, France (V. Bourret, N. Ranc);; ANSES-Nancy, Laboratoire de la rage et de la faune sauvage, Malzéville, France (M. Wasniewski);; Medical University of Gdansk Department of Tropical Parasitology, Gdynia, Poland (M. Grzybek, J. Nowicka, A. Goll);; Université de Montpellier, INRAE, Montferrier-sur-Lez, France (J. Pradel, M. Bouilloud, G. Castel, N. Charbonnel);; University of Antwerp Evolutionary Ecology Group, Wilrijk, Belgium (H. Leirs, V.C. Colombo, V. Sluydts);; Consejo Nacional de Investigaciones Científicas y Técnicas, Buenos Aires, Argentina (V.C. Colombo);; Munster Technological University Department of Biological and Pharmaceutical Sciences, Tralee, Ireland (P. Stuart, A. McManus);; University of Potsdam Institute of Biochemistry and Biology, Potsdam, Germany (J.A. Eccard, J. Firozpoor);; Julius Kühn Institute, Münster, Germany (C. Imholt)

**Keywords:** SARS-CoV-2, COVID-19, Europe, Rodentia, serologic tests, zoonoses, serosurveillance, viruses, coronavirus disease, severe acute respiratory infection coronavirus 2, respiratory infections

## Abstract

We report results from serologic surveillance for exposure to SARS-CoV-2 among 1,237 wild rodents and small mammals across Europe. All samples were negative, with the possible exception of 1. Despite suspected potential for human-to-rodent spillover, no evidence of widespread SARS-CoV-2 circulation in rodent populations has been reported to date.

Esitämme tulokset serologisesta tutkimuksesta, jossa seulottiin SARS-CoV-2 tartuntojen varalta 1,237 luonnonvaraista jyrsijää ja piennisäkästä eri puolilta Eurooppaa. Kaikki näytteet olivat negatiivisia, yhtä näytettä lukuun ottamatta. SARS-CoV-2:n läikkymisen ihmisistä jyrsijöihin on arveltu olevan mahdollista, mutta todisteet viruksen laajamittaisesta leviämisestä jyrsijäpopulaatioissa puuttuvat.

Reverse transmission of diverse zoonotic pathogens (bacteria, viruses, eukaryotic parasites, fungi) from humans to animals has been recognized and documented as a global concern for years (*1*). On July 6, 2022, the World Organisation for Animal Health (OIE) stated, “While occasional occurrences of COVID-19 in domestic or zoo animals show little long-term consequence, infections at wildlife population levels indicate the possibility of further evolution of the virus in animals, and a future reintroduction of the virus into humans at a later date” (*2*). From a One Health perspective, “There is an urgent need to develop frameworks to assess the risk of SARS-CoV-2 becoming established in wild mammal populations” (*3*). In particular, wild rodents are suspected of being among the species more susceptible to SARS-CoV-2 infection, and susceptibility to experimental infection has been confirmed among various rodent species (*4*–*6*). Specific courses of infection may differ among rodent host species, but infection usually results in little or no detectable disease, although infectious virus may shed for 4–7 days after infection and disease may be transmitted to naive rodents (*4*–*6*). These characteristics suggest the potential for reverse transmission, broad circulation, and possible long-term establishment of SARS-CoV-2 in rodent populations. Such an event would be of concern: hamsters, for example, have transmitted SARS-CoV-2 to humans, followed by subsequent person-to-person transmission (*7*). Consequently, on December 6, 2021, the joint United Nations Food and Agriculture Organization and OIE (FAO-OIE) Advisory Group on SARS-CoV-2 Evolution in Animals indicated that a large surveillance study of rodent populations exposed to human contact was needed to close a major gap in SARS-CoV-2 research. 

Animal experiments have shown that antibodies can be detected consistently for several weeks or longer after rodent infection with SARS-CoV-2, although detectable virus shedding lasts only a few days (*4*–*6*). When field prevalence is low or unknown among the target population, serologic testing is the preferred method to maximize chances of detecting circulation of viruses such as SARS-CoV-2 that cause brief infection but maintain longer-lasting serologic response. A recent survey in Hong Kong found a Norway rat (*Rattus norvegicus*) to be potentially seropositive for SARS-CoV-2 (*8*). Considering the high biodiversity and ubiquity of rodents, this finding called for broader surveillance studies in other continents, habitats, and noncommensal rodent species. To investigate its possible reverse zoonotic transmission and establishment in wild rodents in different settings, we conducted a large-scale serologic survey of SARS-CoV-2 in multiple rodent species across Europe. 

We sampled animals in urban parks and zoos, which offer ample opportunity for transmission between humans and rodents, and forests, because other wild forest mammals such as deer have become naturally infected with SARS-CoV-2 (*9*). During 2021, we sampled 1,202 rodents and 35 Soricidae shrews (genera *Sorex* and *Crocidura*) from 23 forests sites and 8 urban parks in 5 countries in Europe (Ireland, Belgium, France, Germany, and Poland) (Figure 1; Appendix 1 Figure 1; Appendix 2). We then assessed each rodent’s SARS-CoV-2 serologic status using an infected cell-based immunofluorescent assay (IFA; Appendix 1) (*10*). We chose the IFA instead of a neutralization assay as the initial screening test because it is scalable to a large number of samples and can be effective in detecting both neutralizing and nonneutralizing antibodies.

All but one of the rodents sampled were IFA negative for SARS-CoV-2. The one IFA-positive rodent (assayed twice on different days to rule out any handling error) was a wood mouse (*Apodemus sylvaticus*) sampled in an urban park near the city of Antwerp, Belgium, on April 6, 2021. We then tested this IFA-positive sample using a seroneutralization assay (Appendix 1), and results were negative, suggesting that the sample had no detectable neutralizing antibodies against the virus strain used in the seroneutralization assay. The sample was also negative by microsphere immunoassay (Appendix 1). The overall serologic status of this wood mouse was therefore unconfirmed. To further investigate possible virus circulation in the area, we used the Luna SARS-CoV-2 RT-qPCR Multiplex Assay Kit (New England BioLabs, https://www.neb.com) to test samples from all 59 rodents captured in the same location as the wood mouse (Appendix 1). PCRs were all negative (including for the IFA-positive wood mouse), which could be expected given the short virus-shedding period described in rodents (*4*–*6*).

Our main conclusion on the basis of this survey is that there is no evidence of a major SARS-CoV-2 spread among wild rodents in northern Europe as of April–September 2021. A similar conclusion had been reached in the study from Hong Kong (*8*), an area with a denser human population and large populations of pest rodents. In that study, serum from 1 urban brown rat was positive in some but not all serologic tests used, and all SARS-CoV-2 PCR tests were negative (*8*). Taken together, these results indicate no evidence of widespread SARS-CoV-2 circulation in rodent populations to date. 

**Figure:**
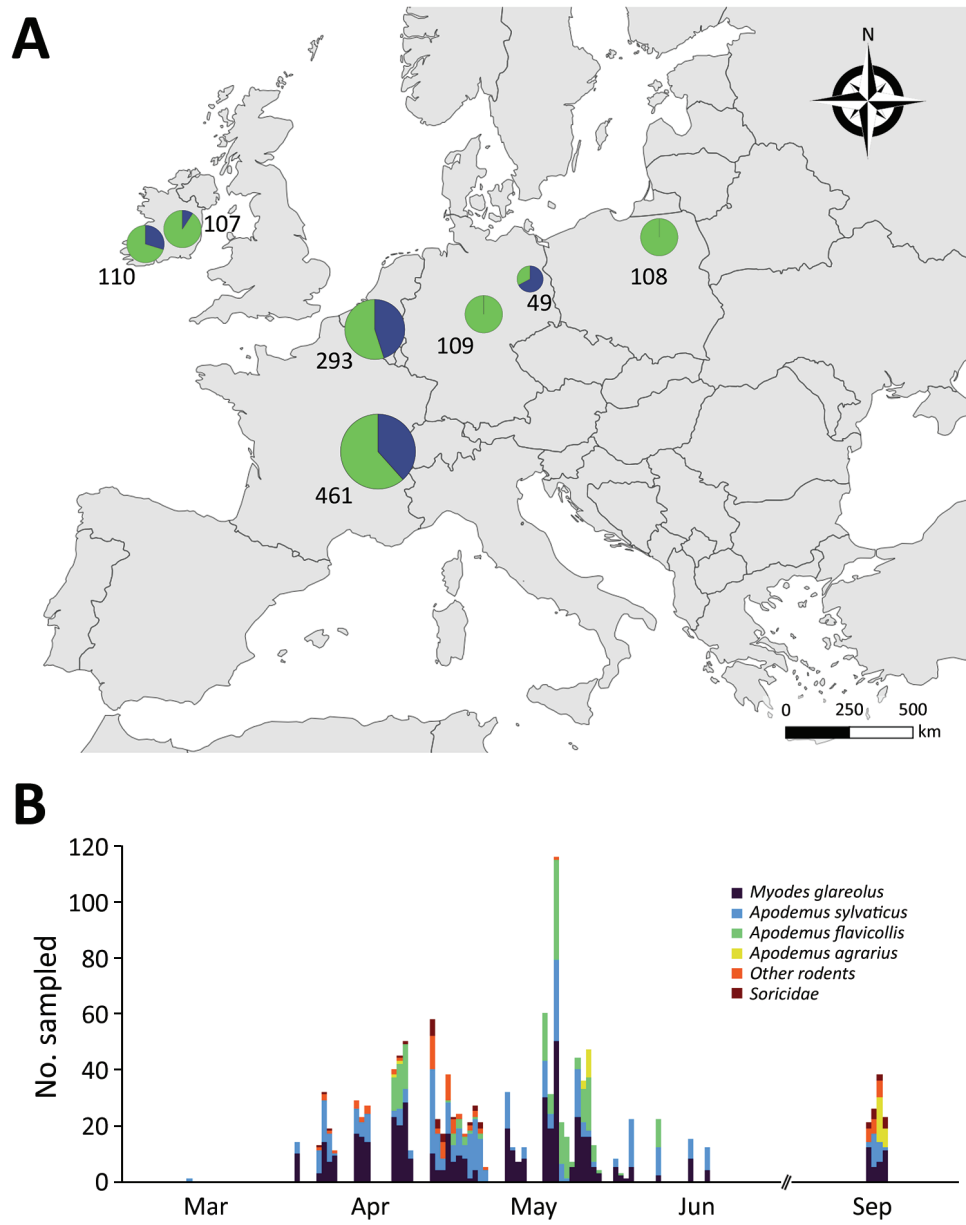
Sampling of various areas in Europe to detect SARS-CoV-2 antibody response in wild rodents. A) Location of sampling areas. Colors indicate the proportion of samples taken in the 2 habitat types (green: forests; blue: urban parks) and symbol size and numbers indicate sample size. Samples were taken from up to 8 different sites in each country (Appendix 1 Figure 1). B) Number of individuals sampled, by date and taxonomy. Details of sampling periods, habitats, and rodent species are provided in Appendix 1 Table 1. Details of sampling periods, habitats, and rodent species are provided in Appendix 2.

Appendix 1 Additional methodologic information about study reporting serosurveillance for SARS-CoV-2 infection among wild rodents in Europe.

Appendix 2 Additional data for study reporting serosurveillance for SARS-CoV-2 infection among wild rodents in Europe.
